# A weather-driven model of malaria transmission

**DOI:** 10.1186/1475-2875-3-32

**Published:** 2004-09-06

**Authors:** Moshe B Hoshen, Andrew P Morse

**Affiliations:** 1 Virtual Population Laboratory, Department of Physics, University of Liverpool, Liverpool L69 7ZE, UK; 2 Department of Geography, University of Liverpool, P.O. Box 147, Liverpool, L69 3BX, UK

## Abstract

**Background:**

Climate is a major driving force behind malaria transmission and climate data are often used to account for the spatial, seasonal and interannual variation in malaria transmission.

**Methods:**

This paper describes a mathematical-biological model of the parasite dynamics, comprising both the weather-dependent within-vector stages and the weather-independent within-host stages.

**Results:**

Numerical evaluations of the model in both time and space show that it qualitatively reconstructs the prevalence of infection.

**Conclusion:**

A process-based modelling structure has been developed that may be suitable for the simulation of malaria forecasts based on seasonal weather forecasts.

## Background

The importance of climate as a driving force of malaria transmission has been known since the earliest days of research on this devastating parasitic disease. However, it is only with the advent of effective weather forecasting techniques that this knowledge may be implemented numerically. Seasonal climate forecasting (with up to six months lead time) has developed rapidly in recent years with a number of atmospheric climate modelling groups showing evidence of skill and reliability in their systems. Because of the chaotic nature of the atmosphere, seasonal forecasts are necessarily probabilistic. These probabilistic predictions are derived from multiple integrations of deterministic climate models. These models successfully predicted the onset and demise of the 1997/1998 El Nino event and its impact on weather in Africa [1]. That event in East Africa was associated with devastating malaria epidemics[2] and, consequently, the health community has shown an increasing interest in the use of seasonal forecasts for predicting epidemics of climate related diseases[3].

The DEMETER project  was aimed to advance the concept of seasonal climate forecasts based on multi-model ensembles. The DEMETER coupled models and the DEMETER retrospective forecast (hindcast) integrations are described elsewhere [4]. The European Centre for Medium Range Weather Forecasting (ECMWF) second-generation global weather re-analysis data set ERA-40,  is being used to test the accuracy ("skill") of the hindcasts. Central to the DEMETER project is an evaluation of the potential of seasonal climate forecasts for end-user communities, such as those concerned with agricultural output and malaria epidemic control[5]. ERA is thus being used as the "gold standard" for the weather forecasts, and in the research presented here is being used as a daily weather database for all Africa.

The aim of the MALSAT group at the School of Tropical Medicine along with the Department of Geography of the University of Liverpool was the assessment of the methodological issues raised by driving a dynamic malaria model with seasonal climate forecasts. In this paper, the first phase is presented, namely the formulation of the model and the development of a dynamic mathematical model of malaria transmission, which can be driven by daily meteorological variables (rainfall and temperature, as provided by ERA-40). Additional research assessed the future risk of malaria epidemics in probabilistic terms[4].

### Mathematical models of malaria transmission

Mathematical models of malaria span nearly a century and are well established. Macdonald[6] reformulated the pioneering model of Ross[7] and identified mosquito vector longevity as the single most important variable in the force of transmission. Further modelling work established vectorial capacity as a practical means of assessing the effectiveness of control measures aimed at the vector[8] and many refinements in modelling technique have since been applied[9,10]. However, these models have, until recently, been dependent on the unrealistic assumption of quasi-static vector numbers[8] and unvarying parasite development rates. Where variation in mosquito numbers has been introduced [11], this was achieved using pseudo-climate, a seasonal variation in mosquito numbers, but not involving variations in vector and parasite development rates, and definitely not in relation to the climate that is actually experienced, i.e. changing weather[12]. In a new report[13], varying mosquito biting frequencies were indeed simulated, but not the co-varying mosquito and parasite weather dependent dynamics.

Climate (as distinct from weather) models of malaria transmission have been developed in recent years to improve our understanding of the likely impact of climate change on malaria transmission. For example, Craig *et al. *[14] developed a fuzzy-logic climate-based distribution model which they suggest could be used to look at the impact of climate change on malaria transmission and, combined with population, morbidity and mortality data, to estimate the burden of disease and aid strategic control of malaria. Lindsay and Birley[15] used a simple mathematical model to look at the effects of temperature on the ability of *Anopheles maculipennis *to transmit *Plasmodium vivax *malaria. Martens [16] used a rules-based modelling approach to examine how climate change might affect global malaria transmission. Lindsay and Martens[17] used a similar model to look at the implications of climate change scenarios on highland malaria in Africa and, more specifically, in Zimbabwe. Hay *et al. *[18] analysed the potential effects of climate change on highland malaria, using a regression approach, and Rogers and Randolph[19] used a statistical model to determine that the global impact of climate change on malaria distribution will be minimal. The relationships identified and applied in the body of research on climate change and malaria transmission highlight the possibility of explicitly relating malaria transmission both spatially and temporally to climatic variables such as temperature, rainfall and (less clearly) humidity. It is, therefore, possible to use these relationships to drive the currently available models of malaria transmission, although, to-date, none of these models are designed to indicate temporal changes in transmission dynamics based on weather[20].

In order to be able to predict within-season and between-year variation in weather-related malaria risk, the model must be driven by varying weather. This paper describes in detail the development of a weather-driven dynamic mathematical malaria model, the final output of which is new infections in the human host. Preliminary results of its numerical evaluation in time and space are presented. The choice of a causal mathematical model rather than a statistical model is based on the knowledge that the former is better suited to extrapolations to novel situations (e.g. when interventions are introduced), and for investigating the non-linear impacts of short-lived changes in driving meteorological determinants. The present malaria model is designed to be used for two distinct but related functions (a) to determine the impact of weather variables on model output (malaria cases/infection) for given interventions and (b) to determine the impact of specific control interventions on model outputs by modifying model parameters.

For the former function, the model can be driven with meteorological variables, from ground-based observations, satellite or modelled weather data, seasonal climate forecasts (and potentially) climate change scenarios. In the second function, the value of a malaria early warning system (MEWS) in terms of triggering earlier or scaling up intervention efforts (e.g. residual spraying) in epidemic years may be assessed. In both cases, the biological processes are included as a series of interlinked sub-models and thus represented as coupled delay differential equations. Each new item of knowledge (such as improvements in the structure of the dynamic equations, due to novel experimental or field data) may be immediately used in the model. New developments such as, for example, the ominous spread of drug resistance may also be immediately incorporated into the model, as, in this example, a reduction in the parasite clearance rate. The implications of all such changes may then be assessed as a quantitative amendment to the prediction. This is done independently for each location and, therefore, the model can be fitted to local conditions, where relevant data are available, or to regional parameters when such data are lacking. Sensitivity analysis can also be used to establish the relative importance of obtaining more accurate data on each parameter

Thus, central to the analysis is the development of a new weather-dependent mathematical dynamic model which no longer attempts to calculate a single constant epidemiological per-case multiplication rate, but follows the temporal progress of the prevalence of infection within a population through seasons and years, To simulate the stochastic elements of the model, delay differential equations based on probabilistic transition between groups rather than on Monte Carlo modelling, as has been undertaken by Gu *et al. *[13], have been formulated..

## Methods

### Data-source

The ERA-40 weather reanalysis data set was chosen because it is the reference data for DEMETER and can provide daily estimates of a range of potentially significant weather variables for the whole globe. This data set was prepared by ECMWF and consists of weather reanalysis data for the whole globe for 40 years (1960–2000) and builds on previous reanalysis data ERA-15 which had been used in an earlier analysis of multi-model ensembles seasonal forecasts . These three- to six-hourly data are stored on the ECMWF site and extracted by local software at the site by the user. Weather variables were extracted from the database as gridded data at one degree (~111 km.) and twelve-hour resolution for the African region. Ideally, daily averaged temperature, accumulated rainfall and humidity would have been the variables. However, reanalysis-based daily average temperature correlated poorly with station data (which is more representative of typical local conditions), and daily minimum temperature proved even worse, and humidity too was poorly modelled. Thus, the analysis used surface (2 m above ground level) daily maximum temperature, offset by -5°C (to roughly represent mean temperature) and total rainfall estimates as input variables. As accumulated rainfall (puddles etc.) is more important than daily rainfall, R_d _(dekadal rainfall), the sum of the previous 10 days of rainfall., were used. There are four malaria parasites (*Plasmodium *spp.), which cause disease in humans. The focus here is on *Plasmodium falciparum*, as it is the principal life-threatening parasite species and is most common in Africa. The temporal resolution of the model was based on the nocturnal activity of the vector and the fact that empirical vector observations are usually made at no better than daily time-resolution. Therefore, the simulation time-step is a single day (24 hours).

It was assumed that the dynamics at the grid-points used do not interact significantly and can be treated as independent, as the distance between grid-points is far greater than the normal mosquito flight distance of roughly 1 to 2 km [21-23]. To simulate large-range transmission by human movement (due to a migrating workforce for example), a small, constant influx of infected people is assumed for each grid point.

### Biological model

Human malaria disease is caused in the individual by an infective mosquito biting a non or semi-immune human. After some two weeks the first gametocytes are produced, independent of ambient temperature. A second mosquito biting the infected human thereafter may ingest gametocytes, which after fertilization pass through the gut wall, develop and ultimately produce sporozoites which become infective when they migrate to the mosquito salivary glands. This process is ambient temperature-dependent. As transmission is less dependent on the number of parasites than on the infective status of the carriers, human and arthropod, only the infection and infectiousness status of the carrier populations are simulated (see Table 1 and 2).

### Modelling the vector population

The most common vector of falciparum malaria in Africa is *Anopheles gambiae (s.l.) *[24,25]. As the female mosquito needs to feed on blood to enable ovum development, its entire life cycle must be modelled. The blood may come also from other mammals, such as cattle (which are not *Plasmodium *hosts), and the mosquito's anthropophilic tendency is an important factor in establishing the intensity of transmission. While the anthropophily varies between regions, at this stage it is assumed constant. The male does not bite and, therefore, does not transmit the disease, and as there are always sufficient males to impregnate the mature females, there is no need to simulate the males' dynamics.

**Figure 1:**
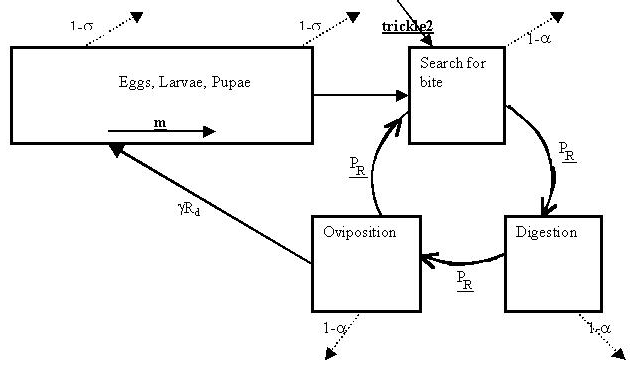
**Schematic presentation of the life cycle *of Anopheles gambiae (s.l.). ***Immature mosquitoes progress at temperature-dependent rate *m*. They are liable to die at daily rate 1-s. Upon completion of immature process they form mature mosquitoes which begin a gonotrophic cycle with progress rate P_R_. They are liable to die at a rate of 1-a per day. New mosquitoes are being imported with rate trickle2. Each mosquito as oviposition lays gR_d _eggs.

The female life is divided into two major parts: the immature stages (egg, larval and pupae), and the mature stage, where onset of maturity is defined as the time of the first flight, which is shortly followed by the first bite. The importance of this division is twofold. First, the immature mosquitoes do not participate in the infection cycle and are, thus, basically in a waiting period, which limits rapid vector population growth. Second, the survivorship (defined as the probability to survive 24 hours) and development rate (part of stage completed in 24 hours) have a different dependence on weather conditions for mature and immature mosquitoes. A schematic representation of the mosquito life cycle is presented in Fig. 1.

### Immature populations

Hitherto, the immature population dynamics have not been involved explicitly or clearly in malaria modelling. The immature forms are water-bound and are thus totally dependent on the existence of water bodies. High temperatures in breeding sites and evaporation (resulting in elimination of puddles, following the cessation of rain) are generally lethal (see below). Unfortunately, reports giving quantified relationships of temperature and rainfall/humidity dependence of the mosquito dynamics are in short supply, although some data are available [26]. Their further publication would assist the development of models.

Eggs are posited by mosquitoes in pools. As a mosquito must find water to reproduce, the oviposition rate is roughly assumed to be proportionate to both the ovipositing mosquito number and to the dekadal (ten-daily) rainfall R_d _filling the pool. Thus a mosquito's probability to oviposit, and for the larvae to survive, is proportional to the amount of water it finds. The inclusion of hydrology and soil typemay improve the understanding of the connection between rainfall and breeding-sites. This dependence on rainfall is valid for areas in which the surface water is dependent only on rainfall. However, in constant mosquito habitats such as stable pools or rice-fields, lack of rain may not limit growth of larvae, but natural predators may curb the growth of larval populations. As the movement between the human location (where a bloodmeal is taken) and the ovipostion site is dangerous and energy consuming, this distance is a major factor in determining the probability of oviposition. Future numeric data for this would be of great value.

There is a shortage of detailed information on the varied survivorship and stage-dependent developmental progress in natural habitats as functions of weather conditions. There are, however, certain limited sources for derivation of information[27,28]. Jepson *et al. *[28] measured the length of each of the three stages (egg L_e_, larvae L_1 _and pupae L_p_) of *An. gambiae s.l. *in 11 different breeding habitats (typically sunlit pools), in which the daytime water temperature was measured. Maturation rate, m, is defined as the fraction of the total immature stage covered in a single day, and is the inverse of the sum of the duration of the immature stages[29,30]

m = 1/(L_e_+L_1 _+ L_p_)     (1).

Thus the maturation rate is a function of temperature for these stages. Even though it is known that high temperatures are detrimental to larvae survival, there are no published numerical data (such as Ndegwa *et al. *[31] found for *Trypanosoma Congolese) *which would allow the introduction of this element.

The most important cause of larval weather-attributable death is probably desiccation[32]. However, in some circumstances eggs can survive for weeks without water[33], and so an immature mosquito rainfall-dependent daily mortality rate, actually resulting in total clearance of the population, is not used in the model. Lack of rain will cause the numbers to be reduced in any case, as above. The overcrowding of immature mosquitoes may result in significant differences in both larval/pupal Survival and also in body size (and thus survival probability of emerging adults). Larval and purpal predation in well-established pools (as opposed to transitory puddles) has significant effects on population development [34]. The model does not currently account for these factors, but assumes a fixed per diem survival rate, s, of 90% [30]. This will be amended, by the aid of new data being collected now, for further model development.

The immature population is, thus, simulated as a set of ν virtual boxes with populations I(n) (n = 1..ν; each box representing the inverse of ν, the length of the immature phase in days). At each simulated day the whole population of each box is multiplied by the *per diem *survival rate σ and moved on by mν boxes (with m, as above, the *per diem *fractional maturation rate). New eggs (box number 1) are laid as a fraction of the number of ovipositing adult females (as shall be discussed). Thus, at each time step, t, the immature population at stage s, I(s,t), has the dynamics:

I(s+mn,t+1) = σ I(s,t)     (2).

Maturing pupae (reaching age n and above) are removed and enter the mature mosquito dynamics according to:



*i.e. *all immature mosquitoes within one day of maturation (at stages above ν(1-m)) will become mature mosquitoes the next day, if they survive(s).

### Mature mosquito dynamics

Mature mosquitoes begin their adult life with their first, nuptial flight, during which fertilization occurs. In the model, a small constant trickle (trickle2) of young uninfected mosquitoes (representing a new imported population) is added to the population of maturing mosquitoes. Afterwards, the development of the fertilized eggs requires the intake of protein, *i.e. *blood meals. Although sometimes more than one initial blood meal is required before egg maturation can occur, and fed mosquitoes that are unable to develop mature eggs are best described as pre-gravid[35], this fraction is neglected at this stage of the model, and all blood meals are assumed to allow, at least potentially, egg maturation. At this level of model development, situations where blood meals are interrupted or when partially fed mosquitoes complete their meal on a second host are ignored. The rate of development of each brood of eggs in a vector is dependent on temperature and, to a lesser degree, on external humidity (probably as a result of the stress caused by a harsher dry environment on the vector). Detinova[36] detected a "degree-day" dependence of the time for the preparation of a brood in *An. maculipennis *(the gonotrophic cycle, G_c_) and hence also of the time for biting, which may be expressed as

G_C _= 1+D_d_/(T-T_c_)     (4);

where D_d _is the number of degree-days required, T_c _the threshold beneath which development halts and T is the daily average temperature. Both D_d _and T_c _are dependent on humidity. In highly humid conditions, D_d _= 37 from Detinova's data. In the tropics G_C _is typically about three days depending on temperature. The temperature dependence of *An. gambiae *is assumed similar until further data become available.

As temperature is not assumed constant (on the contrary, the model is interested in its variation), a daily progress rate (part of gonotrophic cycle covered in one day): P_R _= 1/G_C _is calculated. This assumes that the temperature dependence of the rate is constant throughout the gonotrophic cycle, which is implicit in the degree-day concept.

The completion of a cycle may be established when the sum of daily *P*_*R *_values reaches 1. 37 "boxes" (corresponding to the 37 degree days) are constructed, between which the mosquitoes progress in steps of *P*_*R *_reduced by multiplication by the survival rate (which shall be elaborated below). At the end of a gonotrophic cycle (upon arrival at box 37), each mosquito oviposits and then begins a new cycle. The success of oviposition is dependent on the existence of water-bodies, and hence on dekadal rainfall, and we assume that each ovipositing female lays γR_d _viable eggs, where γ is a constant. The following day these eggs begin the immature mosquito cycle (as above). It seems that the survivorship of mosquitoes is only weakly dependent on their age[6,9,37-39], in spite of some conflicting evidence[40]. The stage most dangerous to the adult mosquito in this model is the feeding stage, consisting of the approach to the mammal for the bite, the duration of the bloodmeal (with the corresponding irritation to the mammal) and the escape to a resting point afterwards. This risk is likely to be increased in unfavourable weather conditions (high temperatures and low humidity), but this has not been investigated yet. Thus, in the present model, the survival of the mosquito per gonotrophic cycle is a constant, a, independent of the duration of the cycle [15]. Estimates of the constant typically vary between 0.4–0.6, and are bound more tightly as 0.48–0.54 by some groups [41-43]. The *per diem *survival is thus calculated by P = α^1/G_c_^. As G_C _is weather-dependent, so is the daily survival. It was assumed that survivorship is independent of the infective state[6,44], even though there are some reports that being infected is harmful to the mosquito.

Combining these, it may be possible to write for δφ, the daily change in the total number of mosquitoes (N_m_) is the difference between the new mosquitoes maturing (and not dying in the period) and the fraction of the mature mosquitoes dying (the daily cycle completion rate 1/G_C _multiplied by the death rate 1-α):



As mentioned, the parasite within-vector dynamics is superimposed on the mosquito dynamics. Mosquitoes are assumed to bite human hosts randomly (independent of their infective status) and thus the proportion of infectious humans (H_i_) rather than non-infectious (H_n_) bitten is the human infectious ratio

r = H_i_/(H_i_+H_n_)     (6).

Non-random biting by mosquito vectors is well described[45] and this could be incorporated in the model at a future stage.

The preference for human biting over cattle is described by the human blood index (B, the proportion of bites on humans, of total bites), which is high (0.6+) for anthropophilic *An. gambiae s.s. *(even though the tendency varies between strains and regions) and generally much lower for zoophilic *Anopheles arabiensis*[46]. Of course, when cattle are far more abundant than humans, the effective B would be reduced. A fraction χ of mosquitoes that bite infective humans become themselves infected and thus the mosquito infection per bite probability is

M_IP _= χ B r     (7)

It is generally assumed that infected mosquitoes stay so for life. The sporogonic cycle (S_C_) (the process of fertilization of the macrogametocyte, formation of the oocyst, ookinete, penetration of the midgut and then the subsequent development of the sporozoites which dwell in the salivary glands) for *P, falciparum *lasts 111 degree-days above 18°C[36]. The daily sporogonic progress (in degree days) is thus S_R _= 111/S_C_. The infectivity of a specific mosquito over its lifetime is dependent on the number of bites it makes after the completion of a sporogonic cycle following the first bite of an infective host.

To combine the gonotrophic and sporogonic processes each of the 37 box-stages of the gonotrophic cycle are sub-divided into 112 sub-sections, numbered 0 to 111, representing progress in degree-days. The 0 subsection reflects an uninfected mosquito. The mosquito population is governed by the following dynamics. An infected mosquito sub-population, M(s,S_s_,t), at stage s of the gonotrophic cycle and at stage S_s _of the sporogonic cycle (in sporogonic-cycle degree days) at time t (in calendar days) progresses each day by gonotrophic rate P_R _and by the sporogonic rate S_R_:

M(s+P_R_S_s_+S_R_,t+1) = pM(s,S_s_,t)     (8).

A finite fraction (1-p) of the mosquito population which dies and thus does not make the transition. Upon completion of the gonotrophic cycle, the process restarts. Upon the completion of the sporogonic cycle the mosquito remains at the infectious stage. If the mosquito is not infected at biting, it remains uninfected throughout the gonotrophic cycle:

M(s+P_R,_0,t+1) = pM(s,0,t) (cycle without infection);     (9a)

but upon biting an infectious human, an uninfected mosquito has a finite probability of either becoming infected

M(P_R_,S_R_,t+1) = pM(0,0,t)M_IP _(new infection)     (9b)

or not:

M(P_R_,0,t+1) = pM(0,0,t)(l-M_IP_).     (9c)

Mosquitoes may arrive at the uninfected biting stage M(0,0,t+1) by two processes, either just after maturation or else by completing an uninfected gonotrophic cycle:



New eggs are laid by mosquitoes completing a gonotrophic cycle:



This means that all mosquitoes located less than P_R _from the end of the gonotrophic cycle will oviposit. Their number must be summed over all infection states 0...111. As discussed above, the average brood size is dependent on rainfall by a multiplicative constant γ. In the present report all the initial mosquitoes were non-infected.

### Modelling the infected host population

Obviously, the focus is on the infected host population dynamics, which reflects the diseased population. The simulation of this population is based on the following assumptions:

All hosts and mature mosquitoes are equivalent except for their infection status. Acquired immunity is not accounted for. Thus, the model reflected the prevalence of malaria infection in the population unless the population is largely non-immune in which case it reflects the prevalence of malaria disease. Immune individuals are assumed to be potential carriers, even though not at personal risk. This issue is contended within the modelling community, and future models will allow for both possibilities.

The crude human death rate is taken as low enough to be unimportant over the time scale. Specifically, the malaria-induced death rate does not influence transmission patterns.

Newly infected patients are not infectious for two weeks, during the intra-hepatic phase of the disease and the early erythrocytic stage, before gametocytaemia rises sufficiently for significant transmission:

H(h+l,t+l) = δH(s,t) (13>h>l)     (12)

Malaria clearance is a slow process. Patients may become uninfected at a constant rate (first order process). The rate selected (δ= 0.97 per day, based on MacDonald's work[6]) enables 90% of the population to clear their infection after 80 days, but other values of lower clearance do not show significantly different results. Super-infection is not accounted for, as for low- to medium-level transmission this effect is of secondary importance, although this may be built into subsequent models. The initial population is assumed to be non-infected, but new infections are being introduced at a constant low rate (*trickle *= 0.01 into a population of 100 every four days). This has the technical benefit of not changing the total population considerably over the simulation period, but limiting the influence of initial conditions found when a large infected population is assumed, or the dying out of the disease after a short initial dry period. This reflects the constant pressure of low-scale transmission by migration (infected migrant workers or troops for instance). This would be quite similar to the case of static communities with low-level external contact [47]. A human bitten by an infectious mosquito may become infected. The finite probability for a bite by an infectious mosquito causing human infection is integrated into the constant c of the reverse process. There is a probability, H_IR_, of a human being bitten by an infectious mosquito each day. This rate depends on the abundance of infectious mosquitoes and of human hosts [48].

There are three components of the human infectious population at time t+1, H(14,t+1): (i) Individuals remaining so from time t, (ii) those who complete the hepatic latent period and (iii) new imports:

H(14,t+1) = (H(14,t)+H(13,t))(δ) + trickle     (13a). 

A host may be uninfected at time t+1, by either (1) remaining uninfected with probability (1-H_IR_), or else (2) being an infected host (S(H(s,t))) and clearing his/her infection with probability (1-δ):

H(0,t+1) = (1-H_IR_)H(0,t)+(1-δ)S(H(s,t))     (13b)

A newly infected host begins the latent phase:

H(1,t+1) = H_IR _H(0,t).     (13c)

The described modelling process was used to establish the fit of the model to a time series of clinical data from Hwange District, Matabeleland, Zimbabwe [49]. Due to the strong dependence of such a local clinical data-set on local weather conditions, the model was driven by station weather data, taken from a CD obtained from NCDC () using Victoria Falls weather station (WMO ID 678430). Weather values for days with missing data (some 10% of all days) were filled by averaging data from adjacent days.

Using the steps described above, the model was run using the ERA-40 weather reanalysis for every grid point covering the African continent (a rectangle from 37°N 18 W to 35 S 52 E, with a mask for areas covering the ocean) over the time period 1987–2000. To allow a spin-up period for the model weather data for 1987 was run twice, while not storing the daily output for the first run, but allowing the first run's output of host, parasite and mosquito situation at the end of the year to be the initial conditions for the "real" run. This allows the simulation of more climatologically realistic starting conditions. Variations in the length of the spin-up period gave similar results. The average prevalence and incidence for the period is then established. Next, the variation of incidence during the period from interannual means was calculated, and hence the standard deviation of annual incidence. This value serves as an estimate of the extent of anomalous malaria, thus reflecting epidemics, beyond holoendemicity and seasonal variation.

## Results

### Development of immature *Anopheles gambiae s.l*

**Figure 2:**
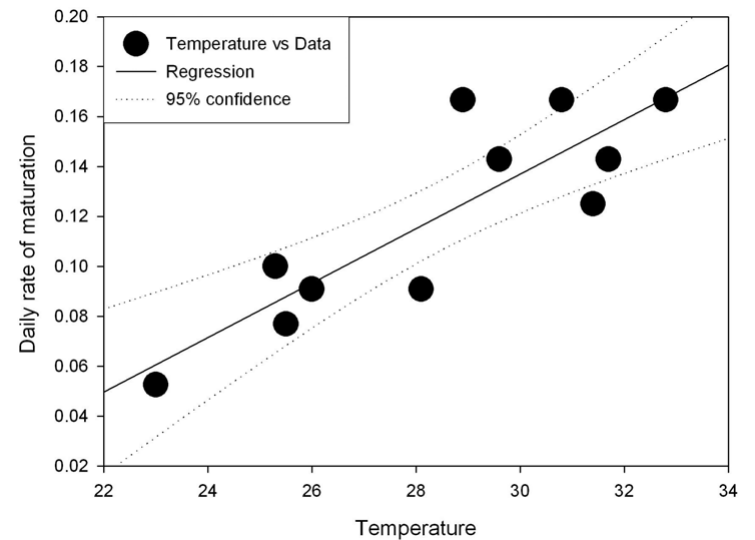
**Larvae maturation. **Rate of development of larvae as a fraction of the complete development cycle as a function of the water temperature in Celsius. Data based on that of Jepson *et al*. Line best fit by least squares. X-axis average water temperature, Y-axis: rate of development (in 1/days) as the reciprocal of length of cycle.

Fig. 2 presents the rate of maturation of larvae using the data from Jepson *et al. *[28]. The x-axis is the average temperature in Celsius and the y-axis is the fraction of the whole larval stage covered in a single day at the given temperature. The straight line is the best-fit (with standard errors)

m = 0.011 (± 0.001) T-0.2 (± 0.26) (1/day)     (14)

Note that the report is for water temperature in shallow pools, which may be significantly higher than the ambient temperature. A few points suggest themselves. To begin with, the intercept with the X-axis is around 18°C. Even though the variation for this value is large, it suggests a lower limit for larval development. Beyond this point, it seems that the assumption in the theoretical methodology of linearity of the development rate with temperature is justified. New data being collected may allow a more thorough validation.

**Figure 3:**
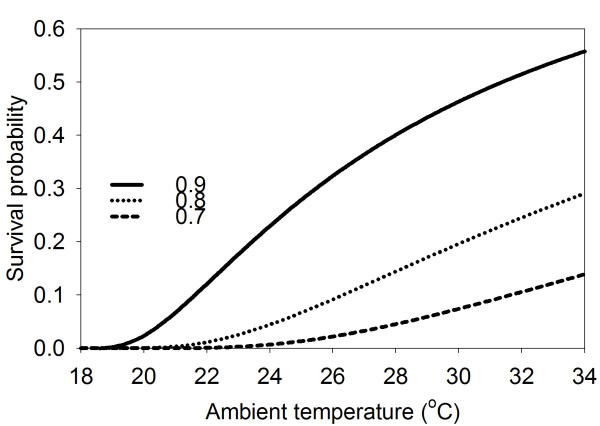
**Larvae survivorship. **The probability of a new *Anopheles gambiae *larva surviving to maturity as function of ambient temperature for different values of *per diem *survivorship. X-axis temperature in °C. Y-axis the probability of completing development until maturity. Lines from top to bottom daily survivorships of 0.9, 0.8 and 0.7 respectively.

Using the proposed rain-dependent daily survivorship (S) and the length of cycle (1/*m*), the per-cycle rain-dependent survivorship is simply S^1/*m*^. The survivorship for the immature stage, by temperature for different values of S, is depicted in Fig. 3. Not surprisingly, larval development increases with rain and temperature. The temperature-linked increase in survival is, however, limited by rainfall. This interdependence of the influence of the two climatic factors limits the regions and times of vector abundance and, hence, also the transmission of malaria. As weather is not constant in reality, numerical integration of the process is required.

### Development of mature *Anopheles gambiae s.l*

**Figure 4:**
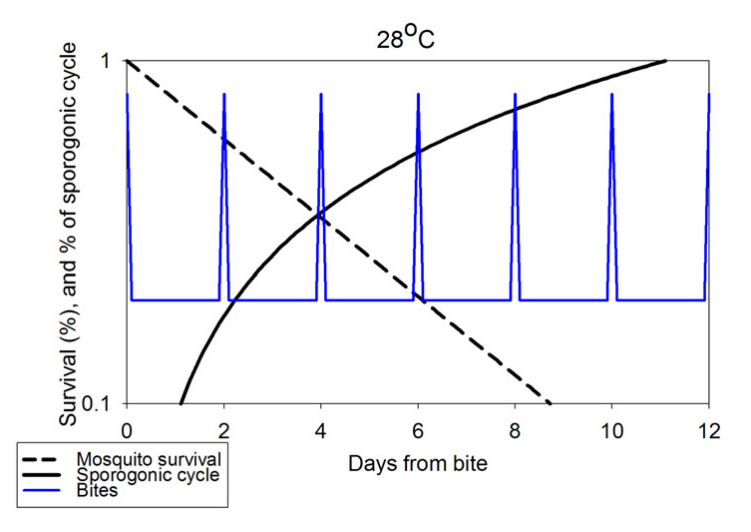
**Vector dynamics and probability of transmission. **The biting cycle (periodic spikes, arbitrary scale), vectorial probability of survival by day (descending line, left axis) and fraction of sporogonic cycle completed (rising line, right axis) for constant temperature. (Ambient temperature 28°C).

**Figure 5:**
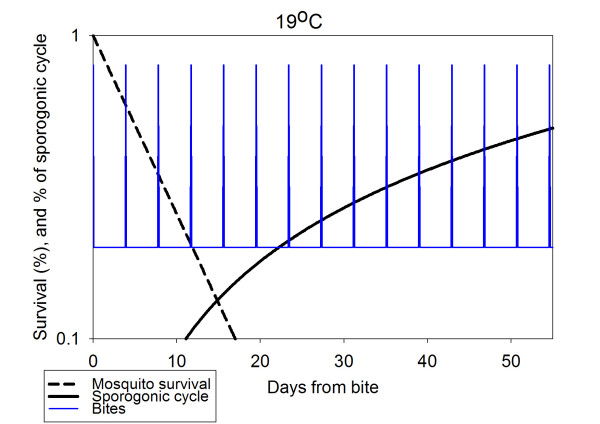
**Vector dynamics and probability of transmission. **The biting cycle (periodic spikes, arbitrary scale), vectorial probability of survival by day (descending line, left axis) and fraction of sporogonic cycle completed (rising line, right axis) for constant temperature (Ambient temperature 19°C)

To stress the importance of the mature-stage dynamics temperature dependence, Figs. 4 and 5 depict the three processes: biting, development of sporozoites within the vector and the vector survival probability as a function of time, for two constant temperatures (assuming humid conditions), 28°C and 19°C. The main points that can be seen from the figures are discussed in turn. At time 0 a female mosquito bites an infected human and begins egg-production, concluding with oviposition. At the end of this process, the gonotrophic cycle, it will bite again and so on as long as it survives. Meanwhile, the mosquito's survival proability drops. Thus the number of mosquitoes which may survive to become infectious from the initial bite at time 0 is decreasing. Meanwhile, the parasites acquired by that initial bite are developing, a process, which may last many gonotrophic cycles. When this process is complete, any surviving mosquitoes become infectious. Thus, the transmission probability is the sum of all survival probabilities after the completion of a sporogonic cycle (when the ascending line reaches 100%). At T = 28°C the sporogonic cycle is completed within less than 12 days, and thus at the next bite over 5% of initial mosquitoes will survive, thus infecting an uninfected human. At T = 19°C the sporogonic cycle lasts for months, and the survival probability of mosquitoes by then is extremely small. This explains the strong transmission in tropical regions and the lack of transmission in temperate zones.

**Figure 6:**
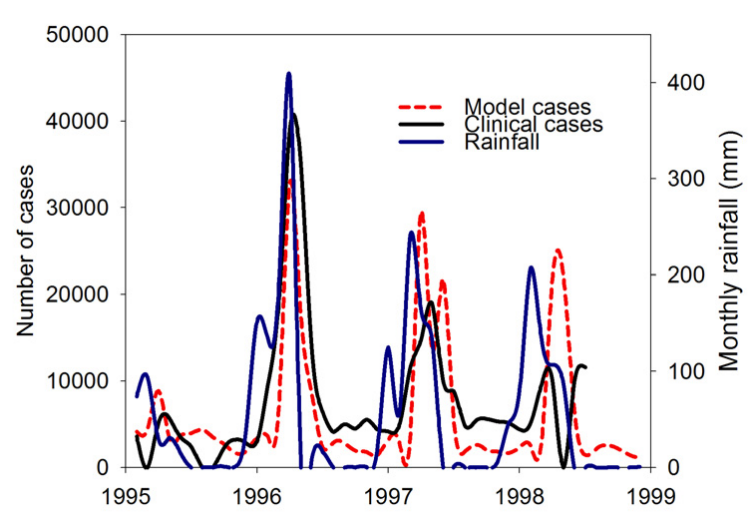
**Rainfall, reported cases and modelled cases for Hwange, Zimbabwe 1995–1998. **Left Y-axis: Case prevalence according to model and according to clinical reports for Hwange, Zimbabwe years 1995–1998^51^. Model results are rescaled. Right Y-axis: rainfall in mm, using station data (NCDC). Blue line Rainfall. Dashed red line Modelled cases. Solid line Reported cases.

**Figure 7:**
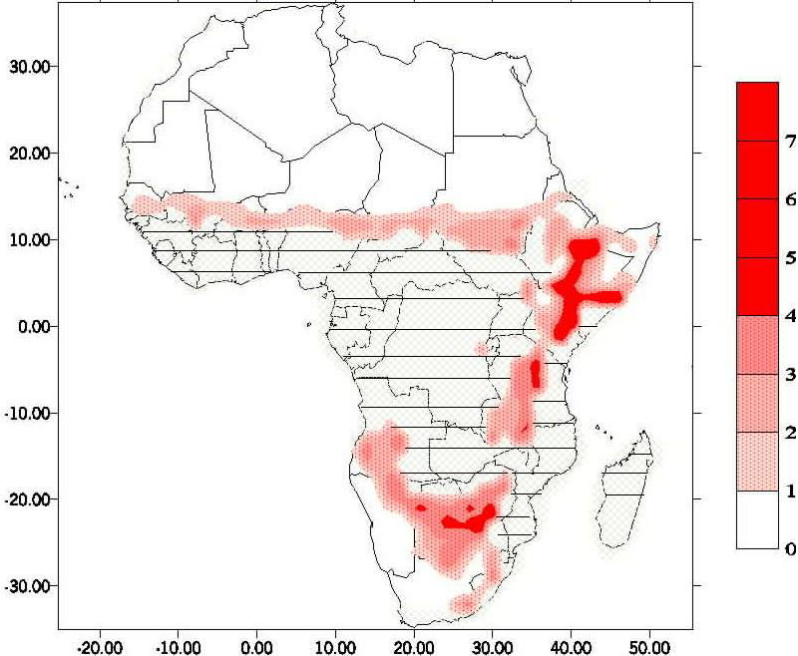
**Spatial epidemicity of malaria. **Interannual standard deviation of incidence of infection as determined from the model run with daily ERA-40 data for the period 1987–2000. Regions with average prevalence rates of >20% (stable malaria) are shaded. Note that since the results are annual averages the values are likely to be less than those recorded from point prevalence surveys during the period of peak malaria transmission.

Fig. 6 shows the fit of the model simulation (based on station data for 1995–1998) to clinical data from Hwange, Zimbabwe[49]. The following graphs are presented: the rain time series, the incidence as calculated by the model and the number of cases of malaria disease as reported by district. In this case we see the main peaks of the 1996–7 epidemic expressed in the model results. It can de seen that the malaria is driven, both according to the model and according to the clinical reports, by the intense rainfall. The rainfall is, however, a local station set, while the malaria clinical results represent a district, in which there was a certain level of prevention and vector control, as well as treatment of cases, all of which prevent the fast exponential increase in case number and predicted by a model, well into the rainy season. Thus, one would expect the model results plot to be spikier than the clinical report. This is, in fact, a general aspect of process-based models, which predict exponential growth of prevalence. The modelling of intervention is an issue under ongoing research. To assess the location of epidemic regions, Fig. 7 presents a map of the anomalous malaria incidence according to the present model for the fourteen years 1987–2000. This was achieved by calculating the interannual epidemic incidence. The anomalies, calculated as standard deviation of annual incidence for each grid-point, are presented in the map. The intensity of spots is proportional to the value of the standard deviation in absolute terms. The regions for which malaria prevalence is usually high (averaging 20%) are blotted out, to differentiate between endemic and epidemic zones. This figure focuses on a different aspect of malaria distribution than usual. The regions in which MEWS will have the greatest benefit are those in which malaria variation is largest. The most pronounced regions are the fringes of endemic transmission. This includes especially the Sahel in West Africa, wide regions of East Africa, ranging deep into Somalia, and in Southern Africa most of Zimbabwe and part of Namibia.

## Discussion

The weather-based dynamic malaria model has been driven here using a reanalysis-climate data set, which is considered to represent the actual historic meteorology (at the appropriate temporal and spatial scale) for the range of climatic variables predicted by the hindcasts in the DEMETER multi-model system. In addition, this reanalysis-based dataset is used to derive the initial conditions of the hindcasts and verification dataset for DEMETER hindcasts. Thus an assessment of the value of the biological malaria model driven by ERA weather data represents the potential value of a "perfect" forecast of climatic conditions to the prediction of malaria epidemics. The assessment of this ability of the weather-driven model to describe interannual variability in malaria infection rates given a perfect forecast, is the basis for a realistic assessment of the benefit associated with the use of operational (and, therefore, unvalidated and imperfect) seasonal climate forecasts in the context of a malaria early warning system.

The dynamic approach claims that total knowledge of the initial state and of the equations, as well as of the external driving forces, allows total knowledge of all future states. However, in real situations, this precise knowledge is often lacking. In many cases, bifurcations of the motion in phase-space due to minute perturbations of initial conditions, may result in large differences in the result, *i.e. *chaos. Chaotic behaviour may, however, be limited by the application of saturation limits to certain model parameters (such as a self-limiting proliferation rate). In addition, the knowledge of the equations themselves is often limited, being typically a linearization of behaviour of empirical data. Thus, there is considerable uncertainty in the result. The dynamic, deterministic method may, however, be used numerically, experimentally, by using small steps so as to validate the stability of the equations. For this reason, the improvement of the understanding of both the ability of weather forecasts to predict weather and of biological models to predict disease, presents a path to the understanding of probabilistic solutions to non-linear epidemic prediction problems.

The capability of seasonal climate forecasts to predict anomalous seasonal climate conditions has improved considerably over the last decade. In particular, the El Niño/La Niña cycle has been correlated with extreme weather conditions throughout the globe[50]. This cycle has been repeatedly related to malaria epidemics. If the existence of an anomalous season can be predicted with skill, and if the relation between anomalous weather and localized malaria epidemics can be determined from time series analysis of a number of events[51], one may assess the probability of unusual increases in malaria transmission resulting from anomalous seasonal climates.

This stage is, however, limited to reconstructions. The good fit in Fig. 2 suggests that the rate of maturation of larvae is linear with temperature. As the measurements were not from a controlled experiment, but from naturally occurring pools, the fit is surprisingly good. Fig. 3 shows the relation between surviving progeny and weather, using rainfall-determined oviposition and the dependence of maturation on temperature. This requires high temperatures (20+ °C) and at least moderate rainfall (10–20 mm/dekad (10 day period)). These conditions suit the regions of known habitation of *An. gambiae s.l. *In some regions, the existence of permanent waterbodies, such as slow-flowing rivers, lakes or swamps may provide suitable breeding sites, and thus make up for the shortage in rainfall, as far as the larvae are concerned. Even though the mature mosquito survival will be considerably reduced by the low humidity[52], malaria incidence during dry seasons will be possible[53]. These effects are not yet implemented in this model.

The requirement for high temperatures for malarial transmission is further illustrated by the plots in Figs. 4 and 5. Even though mosquitoes may well survive and multiply during the summer in temperate regions, they may not become vectors for transmission of *falciparum *malaria, unless the temperature remains in the high 20s (°C) for considerable periods. For this reason falciparum malaria is associated heavily with tropical regions, while in pre-eradication malarious Europe *P.. vivax *was the dominant malaria parasite species.

Further improvements should be added to the model. These may be achieved as new relevant numerical information becomes available on the biological processes, which were here handled somewhat heuristically. For example, the relationship between rainfall and larvae survivorship was simplified due to the lack of data. Laboratory and field, meteorological and entomological data may establish its true form.

The relative importance of the various parameters, assessed by sensitivity analysis by variation of individual parameters is of great interest. This will allow field scientists to focus their efforts in establishing the values of the most critical parameters. In a parallel paper this has been presented. However, a full multivariate evaluation is still underway, using novel parallel computing methods.

The model currently ignores both antiparasitic immunity (immunity to infection) and antitoxic immunity (immunity to disease) – the sharp distinction between which may not exist in reality[54,55]. Neither of these forms of immunity is relevant in areas where 'true' epidemics result from climate anomalies, as it is widely assumed that in these areas the population is largely non-immune and that severe morbidity and mortality due to malaria may occur in all age groups. An age-stratified model is being undertaken at present, but its verification will require considerable input from new targeted longitudinal studies.

However, where the model may be used to predict the impact of an intervention amongst a semi-immune or immune population then both forms of immunity may be significant factors in determining the transmission dynamics of malaria infection[56]. Thus, in endemic areas there will be a need to separate the population between adults and under-fives, and collect suitable data. This work is underway at present.

Numerical evaluations of the model in both time and space show that it has a good first order approximation to the prevalence of infection across the continent. It captures well both the seasonality and interannual variability of infection at the test site in Zimbabwe. Note that there is a large level of under-reporting of clinical cases (due to lack of access to health services) and also over-diagnosis of malaria, which often confound correlations (Barnish, personal communication) [57,58].

The model, when run with the following inputs (Table 1), is able to capture the gross spatial dynamics of malaria transmission across the African continent (Fig. 7). The model represents well both the endemic stable areas of transmission[14], as the shaded regions where average prevalence of infection is above 20%, and the epidemic zones in red, which are not detected by more standard methods. Of course, as with both these previous models, it is clear that there are inherent limitations in trying to fit one model to the entire African continent. In the two examples cited this has been overcome by splicing together two separate models. The development of strain-specific datasets of entomological time-series will provide the basis for separate dynamic models for areas of varying relative abundances of anopheline species. This is one of the reasons for the northern limit of malaria epidemics as depicted being somewhat conservative.

**Table 1:** Values of parameters used

B	0.5
D_d_	37 degree days
H	14 calendar days
M_IP_	0.5
N	37
S_C_	111 degree days
α	0.5.
χ	0.5.
γ	1.0
δ	0.9716.
ν	14.

Stronger variation, both between seasons and interannual, may be expected. Thus malaria transmission will continue further north than suggested, but will also be less stable.

There are other points to consider. The most obvious discrepancy between our results and those of previous workers is that of high prevalence rates being predicted for Somalia – an area where malaria transmission in normally low. The results are created from a relatively short time series and may be disproportionately influenced by the anomalously high rainfall recorded in 1997/1998 in this area, and in East Africa in general, which corresponded with unusually high rates of transmission[3]. The malaria epidemic in southern Africa 1996–7, resulting from the heavy rains during the 1995–6 and 1996–7 rainy seasons in Zimbabwe and its region are reflected by the strong anomaly there. The epidemic character of the East African highlands is well represented too. The southward spread of actual malaria cases, as opposed to model cases, is limited by large-scale prevention schemes in South Africa. Obviously, the crude resolution of the weather data driving the model (approx 111 km^2^) means that the model can only represent the most marked changes in transmission potential over large geographic regions – appropriate to seasonal climate forecasts. This is expressed in the poor spatial resolution as regards Madagascar, in which the spatial variation of prevalence is not represented.

Despite the limitations, which may be overcome in specific regions by using daily weather data at a finer spatial resolution formed by downscaling (a process under considerable meteorological research), the model is capable of describing in general terms the spatial, seasonal and inter-annual variability of malaria transmission in Africa.

The usage of T_max_-5 as a surrogate for daily temperature was an *ad hoc *attempt to create a single value for a wide spectrum varying in time and place. The diurnal variation is, for example, usually far greater in dry regions than in humid conditions. Our attempt was to use a single value which bears relation to something measurable, namelyT_max_. In fact, the two-metre model temperature value, was has been used may not have been the ideal, as larval development is closer to ground, but that would have required detailed soil data, which was beyond the scope of this research. The usage of reanalysis data is obviously inferior to high quality station data. Convective precipitation is highly local and is not well correlated to the averaging required by meteorological reanalysis. For example, the heavy rains in north-western Zimbabwe (end 1995) were under-expressed in ERA-40, partly due to the scale of the reanalysis data, and thus the local malaria incidence using that data is too low. The integration of the data in a reanalysis model in itself makes numerous physical assumptions. In some cases, the result is poor quality of even some large-scale processes, such as the El-Niño of 1997 in East Africa.

High quality station data at the pan-African level is, however, not available. The stations are irregularly dispersed, and not all produce complete data sets, having many missing days. As the model requires daily values, interpolation over large areas with varying orographic characteristics is required with the inherent uncertainty this brings. In addition, the relation between mosquito habitat microclimate and station data too is unknown. High temporal and spatial resolution of weather data will improve the modelling attempts. Among other issues, the continuous measurement of weather data at malaria epidemiological and entomological research and surveillance sites now introduced  will allow development of coupled malaria and meteorological data sets, which will be more effective for future analysis. Development of seasonal climate forecasting tools over large geographic areas, however, will remain for some time partly dependent on verification by reanalysis. Thus, malaria epidemic seasonal early warning will also be linked to this imperfect data source, though perhaps improved by novel downscaling methods.

## Conclusions

This paper presents a first step in the preparation of a weather-driven dynamical model of malaria transmission, for use with both observed weather data and seasonal climate forecasts. The model incorporates the stages of the malaria vector and their dependence on temperature and rainfall, and part of the within-host parasite population dynamics. Some of these elements lack concrete theoretical and empirical development, requiring further input. Further work, under work at present, will enable the employment of such a model in the prediction of outbreaks based on skilful weather forecasts.

## Authors' contributions

MBH formulated the mathematical model, prepared the code and ran the program. APM lead the applications model work package in DEMETER.. Both authors read and approved the final manuscript.

**Table 2:** List of symbols used in text

B	Human blood index, the preference of a mosquito to bite humans and not other animals
D_d_	Length of gonotrophic cycle in degree days
G_c_	Length of gonotrophic cycle in days
H	Hepatic stage in days
H_IR_	Human new infection rate
H_i_	Number of mosquitoes biting infected humans in a day
H_n_	Number of mosquitoes biting uninfected humans in a day
H(s,t)	Human population at stage s of the development of infection at time t. s = 0 symbolises an uninfected host.
I(s,t)	Immature mosquito population at stage s of maturation cycle (in degree days) at time t
L_e_, L_1_, L_p_	Length of egg, larval and pupal stage of mosquito maturation in days
M(s,S_s_,t)	Mature mosquito population which is at stage s of gonotrophic cycle and stage S_s _of sporogonic cycle at time t.
M_IP_	Infection probability of a single mosquito for each bite
m	Maturation rate of larvae in reciprocal days
N	Number of sections into which the gonotrophic cycle was divided.
p	Mosquito population daily survival rate
P_R_	Fraction of gonotrophic cycle covered in one day
r	Fraction of infected humans out of total human population
R_d_	Dekadal (ten daily) rainfall in mm
R_0_	Single case multiplication factor: number of secondary cases induced per case
s	Dummy variable representing stage of development in degree days
S_C_	Length of sporogonic cycle in degree days
S_R_	Daily progression of sporozoites in degree days
S_S_	Stage of sporogonic cycle in degree days. S_S = 0 _represents an uninfected mosquito
T_c_	Threshold temperature for gonotrophic or sporogonic cycle
α	Fractional per-gonotrophic cycle survival of mosquito.
χ	Fraction of mosquitoes biting infective humans that become themselves infected.
γ	Ratio of brood of each ovipositing mosquito to rainfall.
δ	Fractional *per diem *survival rate of human infection. Hence 1-d is the daily infection clearance rate.
φ	Total number of mosquitoes.
ν	Number of sections into which the larval cycle was divided.
σ	*Per diem *larval survival rate
